# Post-operative survival and use of systemic therapy in metastatic long-bone disease: 12 years of institutional experience

**DOI:** 10.1016/j.jbo.2026.100751

**Published:** 2026-02-13

**Authors:** Tom M. de Groot, Joris R.H. Hendriks, Michelle R Shimizu, Jens P. te Velde, Olivier Q. Groot, Erik T Newman, Kevin Raskin, Job N. Doornberg, Paul C. Jutte, Santiago Lozano-Calderón, Joseph H. Schwab

**Affiliations:** a Department of Orthopedic Surgery, Massachusetts General Hospital, Boston, MA, USA; b Department of Orthopedic Surgery, University Medical Center Groningen, Groningen, the Netherlands; c Department of Orthopedic Surgery and Sports Medicine, Amsterdam University Medical Centers, Amsterdam, the Netherlands; d Loyola University School of Medicine, Chicago, IL, USA; e Department of Orthopedic Trauma Surgery, Flinders University, Flinders Medical Centre, Adelaide Australia; f Department of Orthopedic Surgery, Cedars-Sinai Medical Center, Los Angeles, CA, USA

**Keywords:** Metastatic bone disease, Immunotherapy, Targeted treatment, Survival prediction

## Abstract

**Background:**

Survival prediction in metastatic long-bone disease is essential for surgical decision-making, yet the performance of legacy prognostic models has declined in the era of immunotherapy and molecularly targeted agents. As these systemic therapies become routine, treatment patterns themselves may influence survival in ways not captured by earlier models. We, therefore, quantified preoperative systemic therapy use over the last decade and evaluated its association with postoperative overall survival in patients undergoing surgery for long-bone metastases.

**Methods:**

We conducted a retrospective cohort study of 975 consecutive adults who underwent surgery for long-bone metastases at two affiliated tertiary centers between January 2010 and June 2022. Preoperative systemic therapy was categorized into four groups: chemotherapy only, targeted therapy only, combined chemotherapy and targeted therapy, or neither. Postoperative was defined as time from surgery to death from any cause. We described temporal uptake of therapies by calendar year and assessed associations with postoperative survival using multivariable Cox regression adjusting for demographics, ECOG performance status, Katagiri primary tumor grouping, visceral/brain metastases, and laboratory variables. Adjusted survival over calendar time was summarized via marginal standardization at fixed horizons (1, 3, 12, and 24 months).

**Results:**

Use of targeted agents including checkpoint inhibitors rose markedly, from 2% in 2015 to 43% in 2022. In multivariable Cox models, preoperative chemotherapy alone was associated with worse survival versus no preoperative systemic therapy (HR 1.41, 95% CI 1.21–1.65; p < 0.01), as was combined chemotherapy plus targeted therapy (HR 1.24, 95% CI 1.05–1.47; p = 0.01). In contrast, targeted therapy alone before surgery was associated with better survival (HR 0.76, 95% CI 0.58–0.99; p = 0.04). Adjusted survival appeared to increase modestly across calendar years, but the pre-specified joint Wald test for the calendar year spline terms was not significant (p = 0.17), indicating a non-significant trend over time.

**Conclusion:**

This study found that preoperative systemic therapy patterns are strongly associated with post-operative survival in metastatic long-bone disease: targeted therapy alone correlates with improved survival whereas preoperative use of chemotherapy—alone or combined with targeted agents—was associated with worse survival. These findings support incorporating contemporary treatment exposure into prognostic models and suggest that model recalibration to the immunotherapy era may be warranted.

## Highlights

• Preoperative systemic therapy patterns predict survival in long-bone metastases.
• Targeted therapy alone before surgery is associated with improved survival.
• Preoperative chemotherapy, alone or combined, correlates with worse survival.
• Use of preoperative targeted agents for metastatic bone disease increased from 2% in 2015 to 43% in 2022.
• Findings support updating survival models to include systemic treatment exposure.

## 1 Introduction

Metastatic long-bone disease affects a significant portion of patients with advanced stages of cancer, with an estimated prevalence in the United States each year to be up to 400,000 cases [1], [2], [3], [4]. The number of patients with long-bone metastases is expected to increase with improved life expectancy and advancement in cancer therapies [5]. Long-bone metastases contribute to significant morbidity including skeletal related events such as radiotherapy, hypercalcemia, and pathologic fractures, all of which complicate the care and quality of life for cancer patients [6], [7]. Accurate prediction of survival in patients with metastatic long-bone disease is warranted to determine the best course of individual treatment in this complex patient population [8], [9].

One such prediction model, the SORG Machine Learning (ML), was developed for 90-day and 1-year survival of surgically treated patients with long-bone metastases treated from 1999 to 2017 [10]. While the model showed excellent performance on internal and external validations [11], [12], performance was compromised when applied to a more recent patient cohort from 2016 and 2020. In this temporal validation, the model underestimated survival in patients receiving newer systemic agents which were not standard of care during the model-development period and therefore underrepresented in the training data [13].

In the past decade, targeted therapy was introduced as an advanced treatment option due to its high specificity for cancer cells [14], [15], [16], [17], [18], [19]. Checkpoint inhibitors such as PD-1/PDL-1 and CTLA-4 inhibitors have shown great promise in cancer therapy for their ability to reactivate the immune response against tumor cells and thereby improve survival in patients with various types of late-stage cancer [20], [21], [22], [23], [24]. Given the rapid evolution of systemic cancer therapies and increasingly personalized treatment strategies, it is essential that orthopedic oncologists—and the predictive models they rely on—remain aligned with current oncologic practices. When determining the appropriate surgical approach for patients with pathologic fractures, consideration of ongoing systemic treatments and their association with survival is crucial for optimal, individualized care since patients may survive the lifetime of simple osteosynthesis fixation. Access to these novel systemic agents, however, may not be uniform, as insurance status and socioeconomic factors have been associated with disparities in the use of targeted therapies and immunotherapy [25]. Therefore, we aimed to characterize systemic therapy use in two affiliated institutions over a contemporary 12-year period and examine its association with post-operative survival in patients with long-bone metastases. A secondary aim was to examine temporal trends in survival across the 12-year study period. A tertiary aim was to investigate whether uptake of targeted therapy and checkpoint inhibitor monotherapy differed between patients with and without private insurance in our cohort.

## 2 Methods

### 2.1 Setting and participants

Institutional review board (IRB) approval was obtained for this retrospective observational study. This study was performed in accordance with the Strengthening the Reporting of Observational studies in Epidemiology (STROBE) guidelines (Appendix 1) [26].

**Fig. 1:**
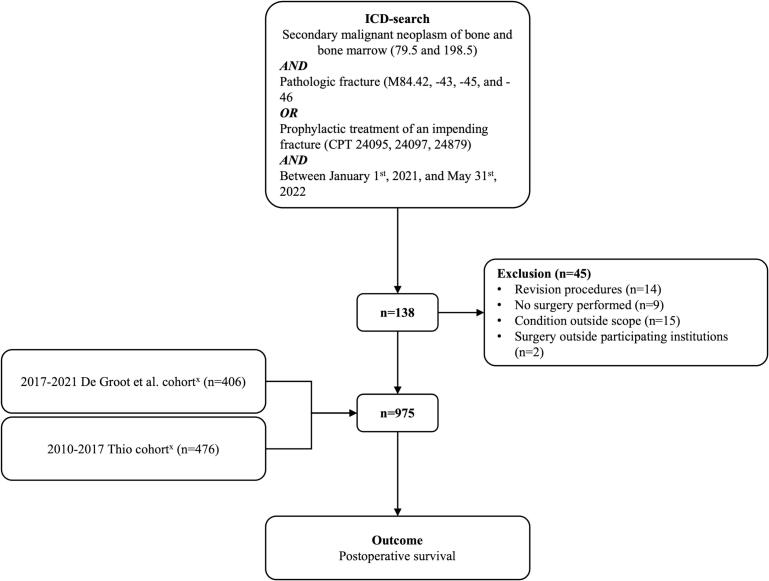
Flowchart of included patients.

Two independent reviewers (TMG, JRHH) manually extracted electronical clinical data from all patients 18 years or older who received surgical treatment for a radiologically confirmed long-bone metastasis at two affiliated tertiary referral urban centers in the United States between January 1st^,^ 2010, and May 31st, 2022. The cut-off of 2010 was chosen because no checkpoint inhibitors were administered before 2010. The year 2022 was chosen to finalize inclusion to have sufficient follow-up time. The cohorts used in the development and temporal validation of the SORG-MLA by Thio et al. (1999–2017), and de Groot et al. (2017–2021) comprising 1,090 adults and 406 patients ≥18 years who underwent surgery for a long-bone metastasis at Massachusetts General Hospital or Brigham and Women’s Hospital were used for the majority of the current study’s dataset [10], [27]. Inclusion criteria were (1) surgical treatment for a metastatic lesion of the long-bones, including patients with malignant lymphoma and myeloma and (2) older than 18 years of age. The exclusion criteria were as follows; (1) patients who received previous surgical treatment for a metastatic lesion of a long-bone and (2) surgical treatment other than intramedullary nailing, *endo*-prosthetic reconstruction, dynamic hip screw, or plate screw fixation. Patients who received multiple surgeries on the same date (e.g., bilateral prophylactic femur stabilization) were also included in the study. These exclusions ensured a homogenous cohort of patients undergoing primary surgical treatment, as prior surgeries such as alternative non-standard procedures, and revision cases could introduce variability in treatment effects, prognosis, and outcomes. A multidisciplinary team of a medical oncologist, anesthesiologist, radiotherapist and orthopedic surgeon were responsible for the assessment of patients’ ability to undergo surgery, and the indication for surgery was set using the Mirels’ criteria as general guideline (Fig. 1) [28].

### 2.2 Outcomes

Our outcome was overall survival, measured from the date of surgery to death from any cause or last follow-up. The last date of follow-up was March 20th, 2025. Of the 975 patients, 884 (91%) were followed for at least 2 years or until death. The remaining 91 patients were lost to follow-up and were censored at their last known contact.

### 2.3 Variables

All targeted therapy agents approved by the Federal Drug Administration (FDA) were included. A list of all included targeted therapy agents can be made available on request.

Our variable of interest was preoperative systemic treatment, categorized as: (1) chemotherapy only; (2) targeted therapy only (including checkpoint inhibitors and other molecularly targeted agents, with selective estrogen receptor modulators (SERMs) recorded separately); (3) both chemotherapy and targeted therapy; and (4) no systemic treatment. For a prespecified subgroup analysis, we restricted to patients who received any systemic therapy preoperatively (chemotherapy and/or targeted therapy).

Other explanatory variables included demographic information such as age, sex, body mass index (BMI), the presence of visceral and brain metastases, as well as the Eastern Cooperative Oncology Group (ECOG) performance score. The ECOG performance score is a widely used measure to evaluate a patient’s level of functioning in terms of their ability to care for themselves, perform daily activities, and physical capacity. It ranges from 0 (fully active) to 5 (deceased) [29]. Performance status was recorded as ECOG and analyzed using three strata: 0,1–2, and 3–4, to reflect the non-linear survival gradient observed in our cohort. Primary tumor histology was documented, and each primary tumor was characterized as being either a slow-growth, moderate growth, or rapid growth type of tumor according to the primary tumor histology grouping of Katagiri et al. (Supplemental Table 1) [30]. In addition, ten preoperative laboratory values were extracted from samples obtained within 14 days before surgery; if multiple values were available, the closest to surgery was used (Table 1).

**Table 1:** Baseline characteristics of included patients (n = 975).

Variables	Total cohort (n = 975)	Neither (n = 372)	Only targeted therapy (n = 49)	Only chemotherapy (n = 298)	Both (n = 256)	*p*-value
	**Median (IQR) | n (%)**					
Age (years)	65 (57–73)	68 (60–76)	66 (58–71)	64 (56–71)	63 (56–71)	**<0.01**
Male sex	443 (45)	203 (55)	27 (55)	116	97	**<0.01**
**Tumor location**						**<0.01**
Femur	713 (73)	254 (68)	31 (63)	223 (75)	205 (80)	
Humerus	207 (21)	93 (25)	16 (33)	61 (20)	37 (14)	
Tibia	32 (3)	15 (4)	2 (4)	9 (3)	8 (3)	
Ulna	3 (0)	2 (0)	0 (0)	1 (0)	1 (0)	
Radius	3 (0)	0 (0)	0 (0)	0 (0)	2 (1)	
Multiple surgeries†	17 (2)	8 (2)	0 (0)	4 (1)	3 (1)	
Pathologic fracture	787 (53)	197 (53)	26 (53)	159 (53)	138 (54)	0.99
**Katagiri tumor group**						**0.03**
Slow growth	380 (39)	122 (33)	14 (29)	133 (45)	111 (43)	
Moderate growth	286 (29)	116 (31)	19 (39)	72 (24)	78 (30)	
Rapid growth	309 (32)	134 (36)	16 (33)	92 (31)	67 (26)	
Visceral metastases	441 (45)	134 (36)	31 (63)	140 (47)	136 (53)	**<0.01**
Brain metastases	163 (17)	45 (12)	16 (33)	50 (17)	52 (20)	**<0.01**
Multiple lesions	778 (80)	268 (73)	253 (84)	42 (86)	215 (84)	**<0.01**
**Laboratory values**						
Absolute lymphocyte count	1.0 (0.7–1.5)	1.3 (0.9–1.7)	1.0 (0.8–1.3)	0.9 (0.6–1.3)	0.8 (0.5–1.2)	**<0.01**
Absolute neutrophil count	5.5 (3.9–7.5)	5.9 (4.5–7.9)	5.2 (3.3–6.0)	5.4 (3.5–7.8)	5.1 (3.4–6.7)	**<0.01**
Albumin	3.7 (3.3–4.0)	3.7 (3.3–4.1)	3.9 (3.5–4.1)	3.7 (3.3–4.0)	3.7 (3.3–4.0)	0.08
Calcium	9.3 (8.7–9.6)	9.3 (8.9–9.7)	9.3 (8.8–9.7)	9.2 (8.7–9.6)	9.1 (8.5–9.5)	**<0.01**
Creatinine	0.8 (0.7–1.0)	0.9 (0.7–1.1)	0.8 (0.7–1.1)	0.8 (0.6–0.9)	0.8 (0.6–1.0)	**0.02**
Hemoglobin	11.2 (9.9–12.4)	11.7 (10.5–13.1)	11.4 (10.5–12.5)	11.0 (9.5–12.1)	10.8 (9.6–11.8)	**<0.01**
Platelet count	250 (186–310)	260 (204–317)	263 (206–321)	247 (176–303)	237 (167–293)	**0.01**
Sodium	138 (136–140)	138 (136–140)	138 (136–139)	138 (136–139)	137 (136–139)	**0.02**
White blood cell count	7.4 (5.5–9.7)	8.2 (6.8–10.8)	7.0 (5.0–8.5)	7.2 (5.0–9.8)	6.8 (4.9–8.3)	**<0.01**
**ECOG-score**						0.06
0	137 (15)	56 (15)	2 (4)	41 (14)	38 (15)	
1–2	703 (72)	257 (69)	40 (81)	218 (73)	188 (73)	
3–4	135 (14)	59 (16)	7 (14)	39 (13)	30 (12)	
**Private insurance (n = 586)**	478 (82)	175 (79)	29 (94)	128 (79)	146 (86)	**<0.01**
**Outcome**						
Overall survival (days)	242 (75–697)	82 (286–873)	425 (208–1143)	184 (75–587)	220 (69–659)	**<0.01**
1-month survival	904 (93)	346 (94)	48 (98)	277 (92)	234 (91)	0.40
3-month survival	717 (74)	285 (77)	42 (86)	215 (72)	175 (68)	0.20
12-month survival	430 (44)	189 (51)	30 (60)	108 (36)	104 (41)	**0.02**
24-month survival	299 (30)	139 (37)	21 (44)	71 (24)	70 (27)	**0.01**

^†^Patients that received surgery for multiple long-bones in one operation session.

^††^Insurance data was missing for more than 35% of patients which was therefore not imputed and used for subgroup analysis.

IQR = interquartile range; ECOG = Eastern Cooperative Oncology Group.

Missing values were present in the following variables: Absolute lymphocyte count: 28.4% (277/975); Absolute neutrophil count: 28.2% (275/975); Albumin: 29.7% (290/975); Calcium: 16.1% (157/975); Creatinine: 13.8% (135/975); ECOG: 21.9% (214/975); Hemoglobin: 12.1% (118/975); Platelet count: 8.0% (78/975); Sodium: 15.9% (155/975); White blood cell count: 12.1% (118/975).

### 2.4 Missing data

Missing data were imputed using the MissForest method [31]. This non-parametric, machine learning technique employs random forest models in an iterative fashion to predict and fill in missing values. By drawing on information from other variables, the algorithm effectively replaces gaps while maintaining the underlying relationships within the dataset. Missing data was present in the following laboratory variables: hemoglobin (8%), platelet count (8%), white blood cell count (8%), sodium (9%), creatinine (10%), calcium (10%), absolute lymphocyte count (21%), ECOG (22%), absolute neutrophil count (22%), and albumin (28%). No missing data was present for the other variables included in this study.

### 2.5 Statistical analysis

We summarized categorical variables as counts (percentages) and continuous variables as mean (SD) or median (IQR), according to distribution assessed with the Shapiro–Wilk test. Overall survival was analyzed with Cox proportional hazards regression. We first ran univariate Cox models for each candidate covariate; variables with p < 0.25 were eligible for multivariable modeling, together with prespecified clinically important factors retained regardless of univariate association [32]. The final multivariable model adjusted for demographics, clinical/treatment factors, and laboratory values, and included calendar year of surgery modeled with B-splines (df = 3) to account for temporal changes in care. Continuous covariates were standardized (z-scores) so that hazard ratios (HRs) were comparable per 1 SD increase. We used a small ridge penalizer and robust (sandwich) standard errors to improve numerical stability. Proportional hazards assumptions were evaluated using Schoenfeld residuals and global tests; no violations were detected (all p > 0.05) [33]. Collinearity was examined with variance inflation factors (no VIF > 10).

To evaluate temporal survival patterns at the population level, we estimated calendar-year–specific adjusted survival probabilities using marginal standardization from multivariable Cox proportional hazards models. For each calendar year, individual survival probabilities at fixed time horizons (1, 3, 12, and 24 months after surgery) were predicted using the spline-modeled calendar year and then averaged across the observed case-mix of the study population. In parallel, we performed a predefined subgroup analysis restricted to tumor types for which molecular targeted therapy is established or commonly applied, using the same modeling and standardization approach [34], [35], [36], [37], [38], [39]. Pointwise 95% confidence intervals were obtained via nonparametric patient-level bootstrapping (1,000 resamples), and evidence for temporal trends was assessed using a joint Wald test of the calendar-year spline terms.

In an exploratory subgroup analysis restricted to patients with available insurance data, we compared the proportions of patients receiving targeted therapy and checkpoint inhibitor monotherapy between those with private versus non-private insurance using χ^2^ tests.

Analyses were performed in Python V3.13 (Python Software Foundation, Wilmington, DE, USA), with the pandas, NumPy, and lifelines packages for survival modeling, and matplotlib for design matrices and visualization. Two-sided p-values < 0.05 were considered statistically significant.

## 3 Results

### 3.1 Patient cohort

We initially identified 1.020 eligible patients operated between January 2010 and June 2022. This comprised 882 patients from earlier work by our study group, and an additional 138 patients from 2021 and 2022 [10], [27]. We excluded 45 patients in total: revision procedures (n = 14), no surgery performed (n = 9), condition outside the scope of this study (patients without confirmed bone metastases, sites other than long-bone, primary bone tumors, osteoporotic stress fracture, chondrosarcoma or acetabular lesion) (n = 15), and surgery outside the participating institutions (n = 2) (Fig. 1).

Of the 975 included patients, the median age was 65 years (IQR 57–73), and 443 (45%) patients were male. The most affected long bones were femur (713/975, 73%), followed by humerus (207/975, 21%) and tibia (32/975, 3%). Tumor growth profiles included 380 patients (39%) with slow-growing tumors, 286 patients (29%) with moderate-growth tumors, and 309 patients (32%) with rapidly growing tumors. Visceral metastases were identified in 441 patients (45%), and brain metastases in 163 patients (17%). Survival at the 1-year point was 48% (473/975) and the median survival was 242 days (IQR, 75–697 days). (Table 1).

### 3.2 Use of systemic therapy

**Fig. 2:**
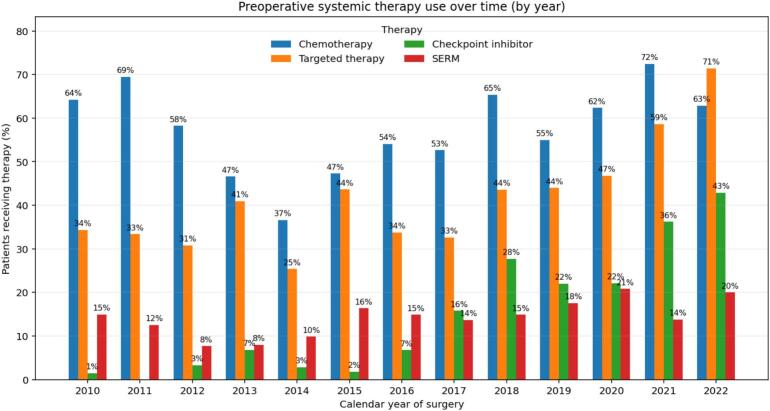
Use of systemic treatment modalities in metastatic long-bone disease over time. SERM: selective estrogen receptor modulator.

Preoperative systemic therapy was administered to 603 patients: 298 (31%) received only chemotherapy, 49 (5%) only targeted therapy, and 256 (26%) received both; 372 patients (38%) received neither. In total, 372 patients (38%) received no preoperative systemic therapy, while 603 patients (62%) did. Among those 603 patients who received systemic therapy, 298 (31%) received chemotherapy only, 49 (5%) received targeted therapy only, and 256 (26%) received both. During the inclusion period, the use of targeted therapy rose steadily from 34% (23/67) in 2010 to 71% (25/35) in 2022. Use of checkpoint inhibitors was negligible early on (<2% before 2013) but increased rapidly from 2016 onward, reaching 43% (15/35) by 2022. Chemotherapy remained the most frequently used modality throughout the inclusion period. Selective estrogen receptor modulator use was consistently low and relatively stable (≈8–20%) (Fig. 2).

In a subgroup-analysis of 586 patients with insurance data, uptake of targeted therapy was higher among those with private insurance (175/478 patients, 37%) than among those without (25/108 patients, 23%; χ^2^ p = 0.008). By contrast, use of checkpoint inhibitors alone did not differ meaningfully by insurance status (99/478, 21% vs. 17/108, 16%; χ^2^ p = 0.24).

### 3.3 Effect of targeted therapy on survival

**Fig. 3:**
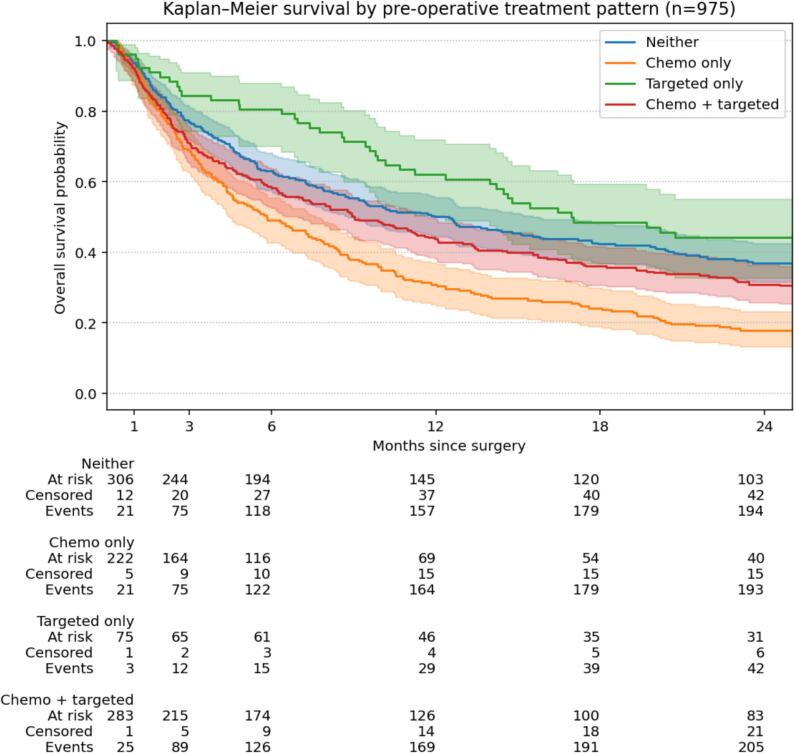
Kaplan-Meier survival curves with 95% confidence intervals illustrating the difference in survival of the treatment groups.

Median survival was 182 days (IQR 65–555) with chemotherapy only, 231 days (77–733) with both therapies, 291 days (92–960) with neither therapy, and 445 days (194–1285) with targeted therapy only. Kaplan Meier survival curves with pairwise log-rank tests with Bonferroni correction showed differences for three comparisons: (1) neither therapy had better survival than chemotherapy only (p < 0.01) and (2) both chemotherapy and targeted therapy (p < 0.01), and (3) targeted therapy showed better survival compared to chemotherapy only (p = 0.03) (Fig. 3).

Compared with neither preoperative systemic therapy, chemotherapy alone was associated with worse survival (HR = 1.41 [95% CI 1.21–1.65]; p < 0.01), as was combined chemotherapy plus targeted therapy (HR = 1.24 [95% CI 1.05–1.47]; p = 0.01). In contrast, targeted therapy alone was associated with better survival (HR = 0.76, 95% CI 0.58–0.99, p = 0.04) (Table 2).

**Table 2:** Cox-proportional hazard regression for overall survival in the full cohort (n = 975).

	*Univariate*		*Multivariate*	
Variable	HR (95%CI)	*p*-value	HR (95%CI)	*p*-value
**Age**	1.07 (1.00–1.13)	0.04	1.06 (0.99–1.13)	0.09
**Male sex**	1.11 (0.98–1.26)	0.09	0.99 (0.86–1.14)	0.84
**Pathologic Fracture**	1.22 (1.07–1.38)	0.00	1.14 (1.00–1.31)	0.06
**Katagiri Tumor Group**				
Slow growth	0.66 (0.58–0.76)	0.00	0.66 (0.57–0.77)	**<0.01**
Moderate growth			Ref	
Rapid growth	1.58 (1.36–1.84)	0.00	1.63 (1.38–1.93)	**<0.01**
**ECOG**				
0			Ref	
1–2	1.44 (1.25–1.65)	0.00	1.25 (1.08–1.44)	**<0.01**
3–4	2.50 (2.02–3.09)	0.00	1.72 (1.37–2.17)	**<0.01**
**Presence of visceral metastases**	1.53 (1.35–1.73)	0.00	1.24 (1.08–1.42)	**<0.01**
**Presence of brain metastases**	1.61 (1.37–1.89)	0.00	1.12 (0.92–1.37)	0.24
**Presence of other bone metastases**	0.93 (0.79–1.08)	0.33		
**Laboratory values**				
Hemoglobin	0.75 (0.68–0.82)	0.00	0.79 (0.73–0.86)	**<0.01**
Platelet count	1.01 (0.94–1.09)	0.70		
Absolute lymphocyte count	0.93 (0.75–1.14)	0.47		
Absolute neutrophil count	1.18 (1.10–1.27)	0.00	1.09 (1.01–1.17)	**0.03**
Creatinine	1.01 (0.95–1.07)	0.82		
White blood cell count	1.13 (1.04–1.22)	0.00	1.04 (0.97–1.13)	0.28
Albumin	0.70 (0.64–0.77)	0.00	0.81 (0.75–0.88)	0.00
Sodium	0.81 (0.75–0.87)	0.00	0.92 (0.86–0.99)	**0.02**
Calcium	1.01 (0.95–1.08)	0.76		
**Treatment**				
Preoperative chemotherapy	1.48 (1.26–1.73)	0.00	1.41 (1.21–1.65)	**<0.01**
Preoperative targeted therapy	0.86 (0.70–1.05)	0.14	0.76 (0.58–0.99)	**0.04**
Preoperative targeted therapy and chemotherapy	1.17 (1.01–1.35)	0.04	1.24 (1.05–1.47)	**0.01**
No preoperative systemic treatment			Ref	

Hazard ratios (HRs) are reported with 95% confidence intervals (CIs). Univariate HRs represent the effect of each variable assessed separately. Multivariable HRs were derived from a Cox proportional hazards model including variables with p < 0.25 in univariate analysis and clinically relevant covariates forced into the model. Calendar year of surgery was modeled using restricted cubic splines to account for temporal trends in treatment and outcomes. Continuous laboratory variables were standardized and modeled per 1-standard deviation increase. ECOG performance status was modeled as a categorical variable (0, 1–2, 3–4), with ECOG 0 as the reference category. Abbreviations: CI, confidence interval; ECOG, Eastern Cooperative Oncology Group; HR, hazard ratio; NSCLC, non-small cell lung cancer treated with molecular targeted therapy.

In a subgroup analysis of 251 patients treated from 2016 to 2020 for which there was preoperative data on tumor response to therapy, targeted therapy alone was associated with improved survival (HR = 0.57, 95% CI 0.38–0.88, p < 0.01), while combined chemotherapy plus targeted therapy was not different from chemotherapy alone (HR = 0.96, 95% CI 0.74–1.24, p = 0.74). Observed tumor response to treatment was strongly associated with better survival (HR = 0.37, 95% CI 0.27–0.50, p < 0.01) (Table 3).

**Table 3:** Cox-proportional hazard regression for overall survival in subgroup of patients with tumor-response data (n = 251).

	Univariate		Multivariate	
Variable	HR (95%CI)	p-value	HR (95%CI)	p-value
**Age**	1.04 (0.92–1.17)	0.54	1.18 (1.03–1.34)	**0.01**
**Male sex**	1.28 (1.00–1.64)	**0.049**	0.93 (0.69–1.25)	0.63
**Pathologic Fracture**	1.31 (1.03–1.67)	**0.03**	1.13 (0.89–1.44)	0.33
**Katagiri Tumor Group**				
Slow growth	0.75 (0.57–0.98)	**0.03**	0.78 (0.60–1.02)	0.07
Moderate growth			Ref	
Rapid growth	1.30 (0.95–1.78)	0.10	1.61 (1.16–2.25)	**<0.01**
**ECOG**				
0			Ref	
1–2	1.30 (0.98–1.74)	0.07	1.24 (0.92–1.68)	0.15
3–4	2.93 (1.97–4.34)	**<0.01**	1.64 (1.10–2.43)	**0.02**
**Presence of visceral metastases**	1.20 (0.94–1.54)	0.14	1.14 (0.88–1.47)	0.32
**Presence of brain metastases**	1.40 (0.98–1.99)	0.06	1.17 (0.82–1.67)	0.39
**Laboratory values**				
Hemoglobin	0.63 (0.56–0.72)	**<0.01**	0.68 (0.60–0.77)	**<0.01**
Platelet count	0.87 (0.75–1.00)	0.054	0.85 (0.74–0.98)	**0.03**
Absolute lymphocyte count	0.98 (0.78–1.23)	0.88		
Absolute neutrophil count	1.15 (0.90–1.46)	0.27		
Creatinine	1.01 (0.94–1.09)	0.71		
White blood cell count	1.18 (1.01–1.39)	0.04	1.20 (1.04–1.38)	**0.01**
Albumin	0.72 (0.60–0.87)	**<0.01**	0.83 (0.71–0.97)	**0.02**
Sodium	0.78 (0.66–0.91)	**<0.01**	0.85 (0.74–0.98)	**0.02**
Calcium	0.99 (0.86–1.15)	0.92		
**Treatment**				
Preoperative chemotherapy			Ref	
Preoperative targeted therapy	0.57 (0.40–0.82)	**<0.01**	0.57 (0.38–0.88)	**<0.01**
Preoperative targeted therapy and chemotherapy	0.84 (0.65–1.08)	0.17	0.96 (0.74–1.24)	0.74
Tumor response to treatment	0.43 (0.33–0.56)	**<0.01**	0.37 (0.27–0.50)	**<0.01**

Hazard ratios (HRs) are reported with 95% confidence intervals (CIs). Univariate HRs represent the effect of each variable assessed separately. Multivariable HRs were derived from a Cox proportional hazards model including variables with p < 0.25 in univariate analysis and clinically relevant covariates forced into the model. Calendar year of surgery was modeled using restricted cubic splines to account for temporal trends in treatment and outcomes. Continuous laboratory variables were standardized and modeled per 1-standard deviation increase. ECOG performance status was modeled as a categorical variable (0, 1–2, 3–4), with ECOG 0 as the reference category. Abbreviations: CI, confidence interval; ECOG, Eastern Cooperative Oncology Group; HR, hazard ratio; NSCLC, non-small cell lung cancer treated with molecular targeted therapy.

To address potential heterogeneity in tumor biology and treatment responsiveness, we performed a prespecified subgroup analysis restricted to tumor types for which molecular targeted therapy is established or commonly applied, including breast cancer, thyroid cancer, non–small cell lung cancer, renal cell carcinoma, melanoma, and multiple myeloma (Supplemental Table 4). Overall, the direction and magnitude of associations for key prognostic variables in this subgroup were largely consistent with those observed in the full cohort (Table 2, Supplemental Table 3).

### 3.4 Survival changes over time

**Fig. 4:**
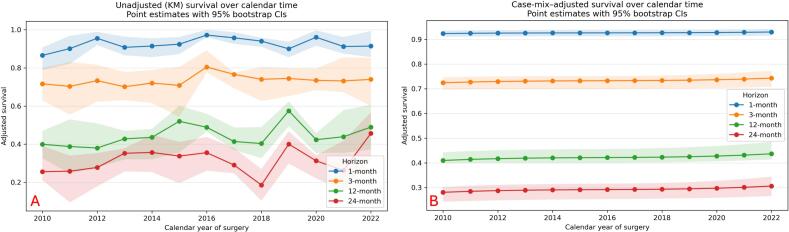
Survival over calendar time unadjusted (A) and case-mix–adjusted (B) for all confounding variables used in the cox-proportional hazard regression models.

Across the 12-year study period, adjusted 1-, 3-, 12-, and 24-month survival probabilities showed a gentle increase over calendar time (Fig. 4). For example, adjusted 12-month survival was 41% (95%CI 39.8% − 44.3%) in 2010 and 43.7% (95%CI 41.4% − 48.5%) in 2022 (Table S4). However, the joint Wald test for the calendar-year spline indicated no evidence of a temporal trend (p = 0.17).

Adjusted 1-, 3-, 12-, and 24-month survival probabilities in tumor subgroups susceptible to targeted therapy demonstrated a similar gradual increase over calendar time (Supplemental Table 4). For example, adjusted 12-month survival increased from 41.0% (95% CI 39.8–44.3%) in 2010 to 43.7% (95% CI 41.4–48.5%) in 2022, while adjusted 24-month survival increased from 28.1% (95% CI 24.3–30.2%) to 30.6% (95% CI 26.6–34.4%) over the same period. Short-term survival showed more modest changes, with 1-month survival increasing from 92.4% to 93.0% and 3-month survival from 72.4% to 74.3%. The joint Wald test for the calendar-year spline showed no statistically significant evidence of a temporal trend in overall survival within this subgroup.

## 4 Discussion

Metastatic bone disease remains a major contributor to morbidity and mortality in advanced cancer. While existing survival models guide treatment planning, our findings highlight how evolving systemic therapies may alter prognostic patterns. Preoperative systemic treatment patterns were strongly associated with survival: patients receiving targeted therapy alone had the longest median survival, while those treated with chemotherapy—alone or in combination—fared worse. Notably, private insurance was associated with use of targeted therapy and survival benefits.

### 4.1 Limitations

This study has several limitations. First, the retrospective design introduces inherent selection bias and limits causal inference. Treatment allocation was not randomized but reflected real-world clinical decision-making in patients with extremity metastatic disease. The population receiving systemic therapy encompasses a wide clinical spectrum, ranging from patients with advanced, treatment-refractory disease to those diagnosed earlier in the metastatic course who may benefit from more aggressive systemic treatment. Although an experimental study design could provide more definitive estimates of treatment effects, such an approach is ethically unfeasible in this context. We therefore categorized patients according to observed preoperative treatment patterns to ensure sufficient statistical power and clinical relevance. However, treatment choices are inherently influenced by patient characteristics—such as performance status, tumor burden, comorbidities, and prior treatment history—which are themselves strongly associated with survival. As a result, the identified treatment groups should not be interpreted as causal effects of therapy but rather as proxies for underlying prognostic profiles, reflecting confounding by indication.

Second, beyond this design-level limitation, the treatment groups differed substantially in baseline disease severity. Patients who received preoperative targeted therapy more frequently presented with visceral and brain metastases and were less often classified as having slow-growth primary tumors according to the Katagiri classification, instead clustering in the moderate- and rapid-growth categories. These differences indicate a higher metastatic burden and more aggressive tumor biology at the time of surgery, which likely influenced treatment selection and explains part of the observed survival differences in unadjusted analyses. Although multivariable models were used to adjust for known prognostic factors, residual confounding from unmeasured variables—such as molecular tumor characteristics or the cumulative effects of prior systemic treatments—cannot be fully excluded.

Third, our study included patients with extremity metastases from diverse primary tumor types, including those with potentially lower chances of success with targeted therapy. This inclusion may have underestimated the impact of targeted treatment on survival, as certain primary tumors are known to respond favorably to targeted therapies. However, the objective of this study was to assess whether treatment patterns could serve as an additional predictive factor for future survival prediction models, rather than directly evaluate the effect of these regimens on survival.

Fourth, this study focused solely on the use of targeted therapeutic agents as a cause for improved survival of patients with long-bone metastases. However, there are numerous reasons for the better survival of these patients such as better preoperative care, earlier diagnosis, and better surgical and anesthetic techniques. However, these factors are difficult to quantify and therefore are hard to implement in predictive models. Together, these observations can introduce some bias on survivorship in this patient population. Nevertheless, the current study highlights the varying effects of different treatment modalities and should be considered as separate variables affecting survivorship in this patient population. Future study focusing more on examining non-clinical sociodemographic variables such as annual household income, presence of a social network, and access to care that may influence the survivorship of surgically treated bone metastases patients is suggested.

Finally, the generalisability of our findings to other settings should be interpreted with caution. Our cohort was derived from a tertiary orthopedic oncology setting with specific referral patterns, insurance structures, and access to systemic therapies, particularly targeted agents. Patterns of systemic treatment use, timing of surgery, and survivorship may differ in other institutions, countries, or health-care systems, especially in settings without private insurance or where healthcare is publicly funded.

This study found that respective treatment modalities being received prior to surgery had varying effects on cancer patients' survivorship. Patients treated with preoperative targeted therapy as a monotherapy showed better survival compared to patients treated with chemotherapy as a monotherapy, patients who had both chemotherapy and patients who have not yet had received systemic treatment at the time of surgery. These findings broadly support the work of other studies in musculoskeletal oncology that evaluate survival after adjusting for patient characteristics, disease progression, growth profile, and treatment factors [40], [41], [42], [43], [44], [45]. Improved survival with novel targeted therapies could be related to their relative specificity to cancer cells. Cancer progression and evasion are commonly attributed to the tumor's ability to manipulate and suppress immune cell activation. The ability of immune checkpoint inhibitors to reverse immunosuppression allows the body to respond more vigorously against cancer cells [43]. On the other hand, targeted therapy identifies and targets genes specific to cancer cells that help tumors grow and change [46]. This specific targeting against cancer cells and its properties contributes significantly to a long-lasting antitumor response and prolongation of survival.

Interestingly, preoperative chemotherapy was associated with worse survival. This concurs with previous studies that have demonstrated a superior response rate and progression-free survival using newer targeted therapies compared to traditional chemotherapy treatment [47], [48]. However, the worse survival outcomes may also reflect the fact that patients who received preoperative chemotherapy were often those with more aggressive or advanced disease. These patients had likely already undergone chemotherapy during earlier stages of their disease but still developed bone metastases, indicating resistance to prior systemic treatments. This suggests that the cancer had progressed despite chemotherapy, representing a more treatment-refractory tumor biology [49]. Consequently, the use of preoperative chemotherapy in these patients may be an indicator of disease severity rather than a causative factor in their poor outcomes.

Accurate prediction of survival in metastatic long-bone disease is essential for both clinicians and patients when deciding together on a treatment plan. The above findings suggest the importance of differentiating between chemotherapy and targeted therapy when electing treatment variables for predictive models. Furthermore, this study’s results highlight the importance of temporal validation as well as the potential of a continuous feedback loop to facilitate self-learning, to maintain a high prediction accuracy for present day and future patients with metastatic bone disease.

Future research endeavors should carefully consider a more detailed selection of treatment related factors when constructing predictive models for survival prediction in metastatic bone disease.

## 5 Conclusion

The findings of this study highlight the importance of differentiating between chemotherapy and targeted therapy when electing treatment variables for predictive models in patients with extremity metastases. Furthermore, patients with private insurance may have had better access to novel targeted treatments for their disease. These findings underscore the need for temporally validated and continuously updated prediction models that can adapt to evolving treatment landscapes.

## Ethics statement

This study was approved by our institutional review board.

## Declaration of generative AI and AI-assisted technologies in the writing process

During the preparation of this work the author(s) used ChatGPT 5.2 to assist with language editing and refinement of the manuscript text. After using this tool/service, the author(s) reviewed and edited the content as needed and take(s) full responsibility for the content of the published article.

## CRediT authorship contribution statement

**Tom M. de Groot:** Conceptualization, Data curation, Formal analysis, Methodology, Visualization, Writing – review & editing, Writing – original draft. **Joris R.H. Hendriks:** Writing – original draft, Data curation, Conceptualization. **Michelle R Shimizu:** Writing – original draft, Investigation, Data curation. **Jens P. te Velde:** Writing – review & editing, Writing – original draft, Investigation, Data curation. **Olivier Q. Groot:** Writing – review & editing, Supervision, Methodology, Investigation, Conceptualization. **Erik T Newman:** Writing – review & editing, Supervision. **Kevin Raskin:** Writing – review & editing, Supervision. **Job N. Doornberg:** Writing – review & editing, Supervision. **Paul C. Jutte:** Writing – review & editing, Supervision. **Santiago Lozano-Calderon:** Writing – review & editing, Methodology, Conceptualization. **Joseph H. Schwab:** Writing – review & editing, Writing – original draft, Supervision, Conceptualization.

## Funding

The authors report no funding disclosures for this study.

## Declaration of competing interest

The authors declare the following financial interests/personal relationships which may be considered as potential competing interests: Tom M. de Groot reports financial support was provided by Foundation De Drie Lichten. Tom M. de Groot reports financial support was provided by Foundation Dr Henry Muller’s National Fund. Tom M. de Groot reports financial support was provided by Vreedefonds. Tom M. de Groot reports was provided by Michael van Vloten Fonds. If there are other authors, they declare that they have no known competing financial interests or personal relationships that could have appeared to influence the work reported in this paper.
