# Supplementary material for: Post-operative survival and use of systemic therapy in metastatic long-bone disease: 12 years of institutional experience
Source: J Bone Oncol. 2026 Feb 13;57:100751. doi: 10.1016/j.jbo.2026.100751 (PMC12925594; doi:10.1016/j.jbo.2026.100751)
Supplement: Supplementary Data 2 [file mmc2.docx]

| **Supplemental table 1. Overview of tumors included** | |
| --- | --- |
| **Primary tumor** | N (%) |
| Breast (hormone dependent) | 179 (18) |
| Prostate (hormone dependent) | 45 (5) |
| Lymphoma | 31 (3) |
| Malignant myeloma | 106 (11) |
| Thyroid | 19 (2) |
| Breast (hormone independent) | 38 (4) |
| Non-small cell lung cancer* | 44 (5) |
| Prostate (hormone independent) | 17 (2) |
| Renal cell | 123 (13) |
| Sarcoma | 27 (3) |
| Gynecological | 17 (2) |
| Other | 20 (2) |
| Other lung | 166 (17) |
| Unknown | 16 (2) |
| Gallbladder | 4 (<0.4 |
| Colon/rectal | 14 (1) |
| Gastric | 2 (0.2) |
| Hepatocellular | 19 (2) |
| Pancreatic | 5 (0.5) |
| Head and neck | 20 (2) |
| Cervical | 0 (0) |
| Urological | 4 (1) |
| Esophageal | 15 (2) |
| Melanoma | 30 (3) |
| **Total** | 975 |
| *Treated with targeted therapy | |

| **Supplementary table 2.** Cox-Proportional Hazards adjusted survival at the 1-, 3-, 12-, and 24-month timepoint. | | | | |
| --- | --- | --- | --- | --- |
| **Calendar year (N)** | **1 month (95%CI)** | **3 months (95%CI)** | **12 months (95%CI)** | **24 months (95%CI)** |
| 2010 (67) | 0.924 (0.911 - 0.936) | 0.724 (0.697 - 0.747) | 0.410 (0.398 - 0.443) | 0.281 (0.243 - 0.302) |
| 2011 (72) | 0.925 (0.912 - 0.937) | 0.727 (0.698 - 0.749) | 0.414 (0.399 - 0.448) | 0.285 (0.246 - 0.307) |
| 2012 (91) | 0.925 (0.912 - 0.938) | 0.730 (0.698 - 0.750) | 0.417 (0.400 - 0.451) | 0.288 (0.248 - 0.311) |
| 2013 (88) | 0.926 (0.912 - 0.938) | 0.731 (0.699 - 0.751) | 0.419 (0.400 - 0.453) | 0.290 (0.250 - 0.313) |
| 2014 (71) | 0.926 (0.912 - 0.938) | 0.732 (0.699 - 0.751) | 0.420 (0.400 - 0.455) | 0.291 (0.252 - 0.314) |
| 2015 (55) | 0.926 (0.912 - 0.939) | 0.732 (0.699 - 0.751) | 0.421 (0.400 - 0.456) | 0.291 (0.252 - 0.315) |
| 2016 (74) | 0.926 (0.912 - 0.939) | 0.733 (0.699 - 0.751) | 0.422 (0.400 - 0.456) | 0.292 (0.253 - 0.316) |
| 2017 (95) | 0.927 (0.912 - 0.939) | 0.733 (0.700 - 0.752) | 0.422 (0.401 - 0.457) | 0.292 (0.254 - 0.317) |
| 2018 (101) | 0.927 (0.912 - 0.939) | 0.734 (0.700 - 0.754) | 0.423 (0.402 - 0.46) | 0.293 (0.255 - 0.319) |
| 2019 (91) | 0.927 (0.913 - 0.940) | 0.735 (0.701 - 0.756) | 0.425 (0.404 - 0.463) | 0.295 (0.257 - 0.322) |
| 2020 (77) | 0.928 (0.913 - 0.940) | 0.737 (0.703 - 0.760) | 0.428 (0.407 - 0.468) | 0.297 (0.259 - 0.327) |
| 2021 (58) | 0.929 (0.914 - 0.941) | 0.739 (0.705 - 0.766) | 0.431 (0.410 - 0.475) | 0.301 (0.262 - 0.334) |
| 2022 (35) | 0.930 (0.915 - 0.942) | 0.743 (0.708 - 0.773) | 0.437 (0.414 - 0.485) | 0.306 (0.266 - 0.344) |
| Adjusted survival probabilities at 1, 3, 12 and 24 months was estimated from a multivariable Cox proportional-hazards model and **marginally standardized**  to the full cohort’s covariate distribution. The model adjusted for age, sex, ECOG (0/1–2/3–4), pathologic fracture, visceral metastases, brain metastases,  laboratory values (hemoglobin, albumin, sodium, WBC, platelets, absolute neutrophils, absolute lymphocytes, creatinine), primary tumor group (Katagiri  categories), and pre-operative systemic treatment pattern; **calendar year** was modeled with **B-splines (3 degrees of freedom)**. Confidence intervals are  **95% percentile bootstrap** (2000 resamples). Patients were **right censored** at last follow-up. | | | | |

| **Supplemental table 3.** Cox-Proportional Hazards regression for tumor subgroups susceptible to targeted therapy. | | | | |
| --- | --- | --- | --- | --- |
| **Variable** | **Univariate HR (95% CI)** | **Univariate *p*** | **Multivariable HR (95% CI)** | **Multivariable *p*** |
| **Age (years)** | 1.02 (0.94–1.10) | 0.67 |  | NA |
| **Male sex** | 0.94 (0.78–1.12) | 0.49 |  | NA |
| **Brain metastases** | 1.71 (1.38–2.12) | <0.01 | 1.10 (0.84–1.42) | 0.49 |
| **Visceral metastases** | 2.05 (1.73–2.43) | <0.01 | 1.46 (1.21–1.76) | <0.01 |
| **Multiple lesions** | 0.70 (0.56–0.86) | <0.01 | 0.89 (0.70–1.11) | 0.30 |
| **Pathologic fracture** | 1.27 (1.07–1.51) | <0.01 | 1.16 (0.97–1.40) | 0.10 |
| **ECOG performance score** |  | |  |  |
| 0 |  |  | ref |  |
| 1-2 | 1.32 (1.10–1.59) | <0.01 | 1.17 (0.97–1.42) | 0.10 |
| 3-4 | 2.28 (1.67–3.12) | <0.01 | 1.62 (1.14–2.30) | <0.01 |
| **Laboratory values** |  |  |  |  |
| Absolute lymphocyte count | 0.80 (0.71–0.90) | <0.01 | 0.93 (0.82–1.05) | 0.25 |
| Absolute neutrophil count | 1.08 (0.96–1.22) | 0.20 | 1.02 (0.88–1.17) | 0.79 |
| Albumin | 0.68 (0.62–0.75) | <0.01 | 0.78 (0.70–0.88) | <0.01 |
| Calcium | 0.99 (0.90–1.09) | 0.85 |  | NA |
| Creatinine | 1.07 (1.00–1.14) | 0.06 | 1.09 (1.02–1.17) | <0.01 |
| Hemoglobin | 0.75 (0.66–0.85) | <0.01 | 0.86 (0.77–0.96) | <0.01 |
| Sodium | 0.86 (0.78–0.95) | <0.01 | 1.01 (0.92–1.11) | 0.86 |
| Platelet count | 0.95 (0.87–1.05) | 0.35 |  | NA |
| White blood cell count | 0.97 (0.88–1.07) | 0.57 |  | NA |
| **Primary tumor** |  |  |  |  |
| Breast cancer (hormone dependent) |  | | ref |  |
| Thyroid cancer | 0.57 (0.45–0.72) | <0.01 | 0.64 (0.50–0.84) | <0.01 |
| Multiple myeloma | 0.88 (0.54–1.42) | 0.59 | 1.00 (0.68–1.48) | 1.00 |
| Breast cancer (hormone independent) | 1.58 (1.11–2.25) | 0.01 | 1.14 (0.80–1.63) | 0.48 |
| NSCLC | 1.27 (0.96–1.68) | 0.10 | 1.38 (1.00–1.90) | 0.050 |
| Renal cell cancer | 1.10 (0.90–1.34) | 0.36 | 1.13 (0.88–1.45) | 0.35 |
| Melanoma | 0.95 (0.62–1.44) | 0.80 | 1.02 (0.67–1.54) | 0.93 |
| **Preoperative systemic treatment** |  | |  |  |
| No preoperative systemic treatment |  | | ref |  |
| Chemo + targeted | 1.56 (1.29–1.90) | <0.01 | 1.27 (1.03–1.57) | 0.02 |
| Chemo only | 1.49 (1.20–1.83) | <0.01 | 1.35 (1.09–1.66) | <0.01 |
| Targeted only | 1.04 (0.75–1.44) | 0.82 | 0.75 (0.51–1.08) | 0.12 |
| Hazard ratios (HRs) are reported with 95% confidence intervals (CIs). Univariate HRs represent the effect of each variable assessed separately. Multivariable HRs were derived from a Cox proportional hazards model including variables with p < 0.25 in univariate analysis and clinically relevant covariates forced into the model. Calendar year of surgery was modeled using restricted cubic splines to account for temporal trends in treatment and outcomes. Continuous laboratory variables were standardized and modeled per 1-standard deviation increase. ECOG performance status was modeled as a categorical variable (0, 1–2, 3–4), with ECOG 0 as the reference category. Primary tumor subtype was modeled using dummy variables, with breast cancer (hormone-dependent) as the reference group. Patients were restricted to tumor subtypes for which targeted therapy is established or commonly applied, including breast cancer, thyroid cancer, non-small cell lung cancer, renal cell carcinoma, melanoma, and multiple myeloma. Abbreviations: CI, confidence interval; ECOG, Eastern Cooperative Oncology Group; HR, hazard ratio; NSCLC, non-small cell lung cancer treated with molecular targeted therapy. | | | | |

| **Supplementary table 4.** Cox-Proportional Hazards adjusted survival at the 1-, 3-, 12-, and 24-month timepoint for tumor subgroups susceptible to targeted therapy. | | | | |
| --- | --- | --- | --- | --- |
| **Calendar year (N)** | **1 month (95%CI)** | **3 months (95%CI)** | **12 months (95%CI)** | **24 months (95%CI)** |
| 2010 (67) | 0.924 (0.911 - 0.936) | 0.724 (0.697 - 0.747) | 0.410 (0.398 - 0.443) | 0.281 (0.243 - 0.302) |
| 2011 (72) | 0.925 (0.912 - 0.937) | 0.727 (0.698 - 0.749) | 0.414 (0.399 - 0.448) | 0.285 (0.246 - 0.307) |
| 2012 (91) | 0.925 (0.912 - 0.938) | 0.730 (0.698 - 0.750) | 0.417 (0.400 - 0.451) | 0.288 (0.248 - 0.311) |
| 2013 (88) | 0.926 (0.912 - 0.938) | 0.731 (0.699 - 0.751) | 0.419 (0.400 - 0.453) | 0.290 (0.250 - 0.313) |
| 2014 (71) | 0.926 (0.912 - 0.938) | 0.732 (0.699 - 0.751) | 0.420 (0.400 - 0.455) | 0.291 (0.252 - 0.314) |
| 2015 (55) | 0.926 (0.912 - 0.939) | 0.732 (0.699 - 0.751) | 0.421 (0.400 - 0.456) | 0.291 (0.252 - 0.315) |
| 2016 (74) | 0.926 (0.912 - 0.939) | 0.733 (0.699 - 0.751) | 0.422 (0.400 - 0.456) | 0.292 (0.253 - 0.316) |
| 2017 (95) | 0.927 (0.912 - 0.939) | 0.733 (0.700 - 0.752) | 0.422 (0.401 - 0.457) | 0.292 (0.254 - 0.317) |
| 2018 (101) | 0.927 (0.912 - 0.939) | 0.734 (0.700 - 0.754) | 0.423 (0.402 - 0.46) | 0.293 (0.255 - 0.319) |
| 2019 (91) | 0.927 (0.913 - 0.940) | 0.735 (0.701 - 0.756) | 0.425 (0.404 - 0.463) | 0.295 (0.257 - 0.322) |
| 2020 (77) | 0.928 (0.913 - 0.940) | 0.737 (0.703 - 0.760) | 0.428 (0.407 - 0.468) | 0.297 (0.259 - 0.327) |
| 2021 (58) | 0.929 (0.914 - 0.941) | 0.739 (0.705 - 0.766) | 0.431 (0.410 - 0.475) | 0.301 (0.262 - 0.334) |
| 2022 (35) | 0.930 (0.915 - 0.942) | 0.743 (0.708 - 0.773) | 0.437 (0.414 - 0.485) | 0.306 (0.266 - 0.344) |
| Adjusted survival probabilities at 1, 3, 12 and 24 months was estimated from a multivariable Cox proportional-hazards model and **marginally standardized**  to the full cohort’s covariate distribution. The model adjusted for age, sex, ECOG (0/1–2/3–4), pathologic fracture, visceral metastases, brain metastases,  laboratory values (hemoglobin, albumin, sodium, WBC, platelets, absolute neutrophils, absolute lymphocytes, creatinine), primary tumor (breast cancer,  lung cancer treated with molecular targeted therapy, renal cancer, thyroid cancer and multiple myeloma) and pre-operative systemic treatment pattern;  **calendar year** was modeled with **B-splines (3 degrees of freedom)**. Confidence intervals are **95% percentile bootstrap** (2000 resamples). Patients were **right**  **censored** at last follow-up. | | | | |


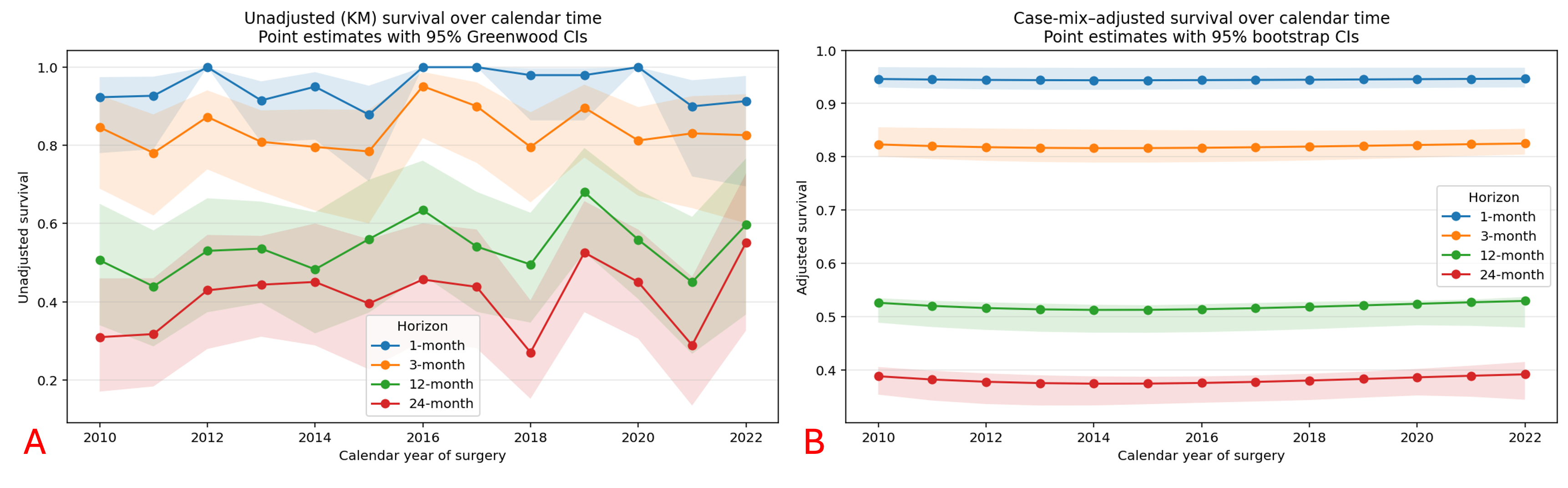


**Supplemental figure 1. Survival over calendar time unadjusted (A) and case-mix–adjusted (B) for all confounding variables used in the cox-proportional hazard regression models for tumors susceptible to targeted therapy.**
